# The contribution of lifestyle coaching of overweight patients in primary care to more autonomous motivation for physical activity and healthy dietary behaviour: results of a longitudinal study

**DOI:** 10.1186/s12966-014-0086-z

**Published:** 2014-07-16

**Authors:** Geert M Rutten, Jessie JM Meis, Marike RC Hendriks, Femke JM Hamers, Cindy Veenhof, Stef PJ Kremers

**Affiliations:** 1 School for Nutrition, Toxicology and Metabolism (NUTRIM), Department of Health Promotion, Maastricht University, Maastricht, The Netherlands; 2 School for Nutrition, Toxicology and Metabolism (NUTRIM), Department of Human Movement Science, Maastricht University, Maastricht, The Netherlands; 3 Public Health Services (GGD) Southern Limburg, Geleen, The Netherlands; 4 Netherlands Institute for Health Services Research (NIVEL), Utrecht, The Netherlands

## Abstract

**Background:**

Combined lifestyle interventions (CLIs) have been advocated as an effective instrument in efforts to reduce overweight and obesity. The odds of maintaining higher levels of physical activity (PA) and healthier dietary behaviour improve when people are more intrinsically motivated to change their behaviour. To promote the shift towards more autonomous types of motivation, facilitator led CLIs have been developed including lifestyle coaching as key element. The present study examined the shift in types of motivation to increase PA and healthy dieting among participants of a primary care CLI, and the contribution of lifestyle coaching to potential changes in motivational quality.

**Methods:**

This prospective cohort study included participants of 29 general practices in the Netherlands that implemented a CLI named ‘BeweegKuur’. Questionnaires including items on demographics, lifestyle coaching and motivation were sent at baseline and after 4 months. Aspects of motivation were assessed with the Behavioural Regulation and Exercise Questionnaire (BREQ-2) and the Regulation of Eating Behaviour Questionnaire (REBS). We performed a drop out analysis to identify selective drop-out. Changes in motivation were analysed with t-tests and effect size interpretations (Cohen’s *d*), and multivariate regression analysis was used to identify predictors of motivational change.

**Results:**

For physical activity, changes in motivational regulation were fully in line with the tenets of Self Determination Theory and Motivational Interviewing: participants made a shift towards a more autonomous type of motivation (i.e. controlled types of motivation decreased and autonomous types increased). Moreover, an autonomy supportive coaching style was generally found to predict a larger shift in autonomous types of motivation. For healthy dietary behaviour, however, except for a small decrease in external motivation, no favourable changes in different types of motivation were observed. The relation between coaching and motivation appeared to be influenced by the presence of physical activity guidance in the programme.

**Conclusions:**

Motivation of participants of a real life primary care CLI had changed towards a more autonomous motivation after 4 months of intervention. Autonomy-supportive lifestyle coaching contributed to this change with respect to physical activity. Lifestyle coaching for healthy diet requires thorough knowledge about the problem of unhealthy dieting and solid coaching skills.

## Background

Combined lifestyle interventions (CLIs) in primary care, including dietary advice and physical activity, have been advocated as an effective instrument in efforts to reduce the growing problem of overweight and obesity [1]–[3]. Enhanced levels of physical activity and a healthier diet maintained over a longer period of time have shown to be associated with better health outcomes for obese individuals [3]–[5]. However, low enrolment rates, high drop-out rates and incomplete implementation have limited the effectiveness of CLIs in real life situations [6],[7]. In contrast to reaching immediate, short-term changes [8]–[10], it has proved difficult to achieve sustained behaviour change, which is required to prevent weight regain and chronic diseases such as type 2 diabetes or cardiovascular diseases among obese individuals [11],[12].

Research has demonstrated that the probability of maintaining higher levels of physical activity and healthier dietary behaviour improve when people are more intrinsically motivated to change their behaviour [13]–[15]. In a recent review on motivation and self-regulation in relation to weight reduction [15], the authors indicated that interventions may so far have focused too much on influencing cognitions and skills and ignored the importance of perceived autonomy in the process of adopting new behaviours [15].

Intrinsic motivation is the most pronounced type of autonomous motivation described in Self-Determination Theory (SDT)[16],[17]. This theory distinguishes 3 types of motivation: amotivation, extrinsic motivation and intrinsic motivation. Extrinsic motivation is subdivided into four types of motivational regulation, viz. two controlled types, external and introjected regulation and two autonomous types, identified and integrated regulation. The theory indicates that the quality of the motivation to engage in a certain behaviour can shift from amotivation and/or more controlled types of motivational regulation towards the autonomous types of regulation and towards the ultimate form of autonomous motivation, intrinsic motivation. To reach this shift, SDT indicates that there are three basic needs, i.e. autonomy, competence and relatedness, that should be supported. If individuals experience an insufficient level of one of these needs it hampers the shift towards autonomous motivation.

To promote the shift in motivation towards the more autonomous types, facilitator-led CLIs have been developed. An example is the Dutch BeweegKuur intervention, which, in addition to physical activity support and dietary advice, includes lifestyle coaching by means of motivational interviewing [18],[19]. Through the combination of these three components the intervention touches on the need for autonomy by means of lifestyle coaching, on competence by means of lifestyle coaching and physical activity and dietary behaviour guidance and on relatedness by means of group sessions. By the inclusion of autonomy supportive lifestyle coaching (LSC), the intervention intends to produce sustainable changes in energy balance related behaviours. The objective of the intervention is to enhance overweight or obese participants’ levels of physical activity and improve their dietary behaviour. The BeweegKuur intervention distinguishes 3 programmes that all include 7 lifestyle coaching sessions and 2 individual and 5 group sessions with a dietician. The programmes differ in the extent and intensity of physical activity support. The Independent exercise programme includes no physical activity support by a physical therapist (PT), while the Startup programme includes six PT sessions in 3–4 months and the Supervised exercise programme includes 3–4 months of intensive PT-guided training at least twice a week. Individuals are assigned to the programmes on the basis of their weight-related health risk, which is based on their BMI, their waist circumference and the presence of risk factors for type 2 Diabetes or Cardio Vascular Disease, or of comorbidities. A low or moderate level of physical activity is also an inclusion criterion for the BeweegKuur intervention. The LSC carries out the primary assessment and includes people in the intervention. LSCs involved in the BeweegKuur intervention, which in most cases are general practitioner assistants and sometimes physical therapists, are trained in motivational interviewing (MI), a method for autonomy-supportive coaching [20],[21].

In their review, Teixeira et al. [15] demonstrate that, despite the importance of autonomous motivation for sustained behaviour change and despite the fact that it is the primary focus of its application, few studies on motivational interviewing and weight loss have considered changes in the quality of motivation as an outcome.

The present study aimed to assess the shift in quality of motivation to increase physical activity and to engage in a healthier diet among participants of the BeweegKuur intervention. In addition the study aimed to examine the contribution of lifestyle coaching to potential changes in motivational quality. Given the autonomy supportive character of MI, it was expected that the MI aspects of the LSC’s counseling style would reduce controlled types of motivation and increase autonomous types of motivation.

## Methods

### Design and recruitment

In this prospective multicentre cohort study, data were gathered from a sample of 29 out of 150 BeweegKuur locations. The 29 locations were spread geographically across the Netherlands. Data were collected using a longitudinal questionnaire survey with two measurements: at baseline and at 4-months follow-up.

All locations included a general practitioner (GP), a GP assistant, a physical therapist and a dietician. The GP preselected potential participants and referred them to the LSC. The LSC had one to three sessions with the participant before the latter made a decision on whether to enroll in the BeweegKuur intervention. LSCs were allowed to include a maximum of 20 participants for every programme. After the participant had given informed consent, the LSC handed them the baseline questionnaire. Further questionnaires were sent to participants directly by the researchers by mail, while a web based version was available for those who preferred it. To reduce the rate of non-response, participants were reminded after two weeks by email and after 4 weeks by phone, or by another email if they were not reachable by phone. The study was approved by the Medical Review Ethics Committee (MEC) azM-UM (File no. NL 32615.068.10/MEC 10-3-051).

### Measurements

#### Background variables

The questionnaires assessed demographic variables (age and gender), educational level (high, medium, low) and employment status (paid job or not). Self-reported BMI was established by asking participants to fill in their body length and weight.

#### Motivational regulation

The quality of motivation for physical activity was assessed using the Behavioural Regulation and Exercise Questionnaire (BREQ-2) [22], including four complementary items on integrated regulation [23]. Answers were given on a 5-point Likert scale (strongly disagree to strongly agree). Cronbach’s alpha reliability coefficients for the BREQ-2 subscales in the present study varied from 0.73 to 0.91. In accordance with a previous study [24], the item ‘Because I get restless if I don’t exercise regularly’, was removed from the identified regulation subscale, since reliability analysis indicated that it detracted from the internal consistency of the subscale.

Quality of motivation for healthy nutrition was measured with an abbreviated 12-item Dutch version of the Regulation of Eating Behaviours Scale (REBS) [25]. All motivational regulations were measured by 2 items with a 5-point Likert answering scale (strongly disagree to strongly agree). Cronbach’s alpha reliability coefficients for the REBS subscales in the present study varied from 0.61 to 0.84.

#### Lifestyle coaching

Participants’ experiences with the LSC were assessed by means of questions developed using the BeweegKuur LSC protocol as a lead [26]. This protocol describes the actions the LSC is expected to perform and includes a manual for motivational interviewing. Questions were developed by the primary research group and reviewed by members of the advisory board, after which the questions were adjusted in accordance with the comments. This resulted in 21 questions concerning the LSC, 10 of which concerned concrete actions, e.g. ‘performed body measurements’ or ‘referred to physical therapist’, and 11 concerned the LSC’s communication style, e.g. ‘made me realize that my diet is unhealthy’ or ‘took the decision to participate in the BeweegKuur for me’. Answers were scored on a 5 point Likert scale (completely disagree to completely agree). A principal component analysis (oblimin rotation, 25 iterations) revealed that participants had experienced 3 main categories in coaching styles (See Additional file 1). Three types of lifestyle coaching were distinguished: *autonomy-supportive*, in which the LSC helps and supports the participants in making their own decisions (7 items, Cronbach’s α = 0.74), *controlling*, in which the LSC makes the decisions for the participants (2 items, Cronbach’s α = 0.66), and protocol adherent, which means that the LSC applies the tests recommended in the protocol (6 items, Cronbach’s α = 0.65).

### Drop-out analysis

Binary logistic regression analysis with drop-out as the dependent variable (non drop-outs = 0, drop-outs = 1) was used to identify selective drop-out. The demographic variables gender, age and educational level were included as covariates, together with the programme variables and the types of motivation for physical activity and healthy diet.

### Data analysis

Prior to the analyses, the database was checked for outliers and missing data and ‘cleaned’. In case of anomalous data (for instance multiple boxes checked were only one was requested), the questionnaires were checked again, and if that did not lead to greater clarity, the item was scored as missing. Parametric tests were only performed if data had a normal distribution. In the various analyses missing data were handled as system missing. In general, analyses included 255 to 290 (>85%) of 298 participants due to missing data, showing that the amount of missing data was limited.

We used descriptive analysis for the demographic variables. Differences in scores for the various types of motivational regulation between programmes at baseline were analysed with one-way ANOVA with post-hoc analysis. T-test for dependent samples was used to assess changes in motivation between baseline and 4 months follow-up. Changes were expressed in effect sizes (ES) using Cohen’s d. In accordance with Cohen’s classification [27] we categorized an ES of 0.2 as small, ES of 0.5 as medium and ES ≥ 0.8 as large.

Relations between types of lifestyle coaching and changes in motivational regulation were analysed with multiple linear regression analyses (backward method). The types of motivation (i.e. amotivation, external, introjected, identified and integrated regulation and intrinsic motivation) were subsequently included as the dependent variable in the analyses. The demographic variables gender, age and educational level, lifestyle coaching, and the motivation scores at baseline were included in the model**.** Since participants were assigned to one of the three BeweegKuur programmes that differed in their physical activity guidance, we also added the BeweegKuur programmes as dummy variables to the equation (with the Independent exercise programme as reference). After interpretation of the results of these analyses we explored the possibility of interaction between programme and autonomy supportive or controlled coaching. For that purpose we added four interaction terms (programme * coaching) to the equation. All analyses were performed with SPSS 20 for Windows with α = 0.05.

## Results

### Characteristics of participants

At baseline 409 participants were included, 72.9% (n = 298) of whom also completed the follow-up questionnaire. The average age of these 298 participants was 55.3 years old (SD = 12.2) and 64.8% were female. The mean BMI was 32.9 kg/m^2^ with 73.0% of the participants being obese. A low educational level was reported by 35.9%, a high level by 22.4%, and 47.3% had a paid job. ANOVA tests showed no significant baseline differences between participants of the three different programmes as regards types of motivation for physical activity (0.208 ≤ p ≤ 0.945) or for healthy dietary behaviour (0.091 ≤ p ≤ 0.993).

### Drop-out

The results of the drop-out analysis showed that neither the demographic variables, nor the programme that participants were assigned to, were predictors of drop-out. Baseline types of motivation for physical activity were not significantly related to drop-out either. As regards healthy diet, a higher level of integrated motivational regulation at baseline predicted a lower odds of drop-out (OR = 0.66, p = .037).

### Motivation for physical activity and healthy dietary behaviour

**Figure 1:**
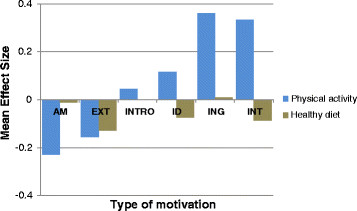
Changes in types of motivation for physical activity and healthy diet.

Amotivation (ES = ^-^0.23) and external regulation (ES = ^-^0.16) for physical activity decreased significantly between baseline and 4 months (Table 1) with a small effect size. Integrated regulation and intrinsic motivation increased, with a small to moderate effect size (ES = 0.36 and 0.33, respectively; Figure 1). As regards healthy dietary behaviour, only external motivation decreased significantly, with a small effect size (ES = −0.13; Figure 1). All other types of motivation did not significantly change (Table 1).

**Table 1:** Changes in motivation for physical activity and healthy diet between baseline and 4 months n ≥ 279; T1 = 4 months

**Physical activity**	**Mean difference T1-T0**	**SD**	**95% CI**	**t**	**P**	**Adj. mean difference T1-T0**	**SD**	**P**
intrinsic motivation	0.328	0.908	0.223 ; 0.433	6.131	.000	0.350	0.130	.000
integrated regulation	0.344	0.899	0.239 ; 0.449	6.425	.000	0.355	0.160	.000
identified regulation	0.078	0.733	−0.007 ; 0.164	1.823	.071	0.073	0.083	.000
introjected regulation	0.051	1.180	−0.087 ; 0.189	0.731	.466	0.050	0.265	.002
external regulation	−0.141	0.954	−0.252 ; −0.030	−2.507	.013	−0.131	0.202	.000
amotivation	−0.188	0.960	−0.300 ; −0.075	−3.287	.001	−0.164	0.185	.000
**Healthy diet**								
intrinsic motivation	−0.084	0.811	−0.178 ; 0.010	−1.750	.081	−0.086	0.146	.000
integrated regulation	0.009	0.845	−0.090 ; 0.107	0.175	.861	0.002	0.157	.860
identified regulation	−0.049	0.745	−0.135 ; −0.038	−1.109	.268	−0.035	0.105	.000
introjected regulation	−0.002	1,108	−0.131 ; 0.127	−0.027	.979	0.018	0.068	.000
external regulation	−0.147	1.087	−0.273 ; −0.020	−2.284	.023	−0.131	0.160	.000
amotivation	0.011	0.889	−0.093 ; 0.114	0.200	.841	0.017	0.177	.111

### Longitudinal relation between lifestyle coaching and motivation

#### Physical activity

As regards physical activity, a higher age predicted higher identified and integrated motivation (Table 2). A higher educational level predicted lower amotivation and lower introjected motivation at 4 months.

**Table 2:** Longitudinal relation between lifestyle coaching and motivation for physical activity

	**Amotivation**			**External regulation**			**Introjected regulation**			**Identified regulation**			**Integrated regulation**			**Intrinsic motivation**		
Independent variables	B	β	*P*	B	β	*P*	B	β	*P*	B	β	*P*	B	β	*P*	B	β	*P*
Constant	1.267		.000	−0.375		.273	1.425		.000	1.105		.001	1.077		.000	0.475		.193
***Demographic variables***																		
Gender																		
(0 male, 1 female)																		
Age (Years)										0.008	0.148	.008	0.010	0.127	.012			
Educational level (Low, Med, High)	−0.146	−0.164	.006				−0.223	−0.147	.009									
***Lifestyle coaching***																		
Autonomy supportive	−0.178	−0.153	.009	0.163	0.115	.039				0.139	0.121	.030				0.206	0.128	.010
Controlled	0.122	0.174	.004							−0.122	−0.177	.001	−0.105	−0.109	.030			
Protocol adherence																		
***Programmes***																		
Startup programme	−0.328	−0.204	.005							0.249	0.158	.020						
Supervised programme										0.232	0.175	.010				0.230	0.124	.043
***Baseline motivation***																		
Corresponding motivation at baseline	0.240	0.302	.000	0.402	0.453	.000	0.466	0.451	.000	0.380	0.403	.000	0.543	0.588	.000	0.532	0.590	.000

n = 255.

Autonomy-supportive lifestyle coaching for physical activity predicted lower amotivation and higher external, identified and intrinsic motivation. Controlled lifestyle coaching predicted higher amotivation and lower identified and integrated regulation. Higher levels of protocol-adherent care by the LSC did not predict any change in the type of motivation.

Being included in the Startup exercise programme predicted lower amotivation and higher identified motivation at 4 months, whereas the Supervised exercise programme predicted higher identified and higher intrinsic motivation.

### Interaction between programme and type of coaching for physical activity

We observed significant interaction effects on integrated motivation between the Start-up programme (p = 0.031) as well as the Supervised exercise programme (p = 0.007) and controlled coaching. An interaction effect was also observed for the Start-up programme with controlled coaching on identified motivation (p = 0.049). For all other interaction terms, no significant effects were found. Our subsequent analyses with stratification for programme revealed that higher levels of perceived controlled coaching predicted a lower level of integrated motivation for the Independent exercise programme (p = 0.001) and a lower level of identified motivation for the Independent (p = 0.041) and Supervised exercise programme (p = 0.010).

### Healthy diet

As regards healthy diet, we found no relations between lifestyle coaching and any type of motivation for healthy diet. A higher educational level and participating in the Start up or Supervised exercise programme predicted lower amotivation (Table 3). Participating in these exercise programmes also predicted higher integrated motivation.

**Table 3:** Longitudinal relation between lifestyle coaching and motivation for healthy diet

	**Amotivation**			**External regulation**			**Introjected regulation**			**Identified regulation**			**Integrated regulation**			**Intrinsic motivation**		
Independent variables	B	β	*P*	B	β	*P*	B	β	*P*	B	β	*P*	B	β	*P*	B	β	*P*
Constant	1.466		.000	1.116		.000	1.089		.000	1.546		.000	1.160		.000	0.802		.000
***Demographic variables***																		
Gender																		
(0 male, 1 female)																		
Age (Years)																		
Educational level (Low, Med, High)	−0.244	−0.237	.000															
***Lifestyle coaching***																		
Autonomy supportive																		
Controlled																		
Protocol adherence																		
***Programmes***																		
Startup programme	−0.364	−0.197	.005										0.343	0.164	.011			
Supervised programme	−0.292	−0.188	.007										0.252	0.143	.026			
***Baseline motivation***																		
Corresponding motivation at baseline	0.325	0.323	.000	0.464	0.527	.000	0.501	0.517	.000	0.297	0.312	.000	0.527	0.557	.000	0.670	0.637	.000

n = 255.

### Interaction between programme and type of coaching for healthy diet

There was an interaction effect of the Start-up programme (p = 0.002 to 0.046) as well as the Supervised exercise programme (p = 0.004 to 0.021) and controlled coaching for all three types of autonomous motivation for healthy eating. We also observed an interaction effect of the Start-up programme and controlled coaching for introjected (p = 0.019 ) and external motivation (p = 0.008) and of the Supervised exercise programme and controlled coaching (p = 0.006) on amotivation. In the stratified analyses higher perceived controlled coaching predicted a lower level of intrinsic (p = 0.014), integrated (p ≤ 0.001) and identified motivation (p = 0.002) for healthy eating of participants assigned to the Independent exercise programme. Higher levels of controlled coaching also predicted a higher external motivation for participants in the Start-up programme, and a higher level of amotivation for the Independent exercise (p = 0.001) and Start-up programme (p = 0.035).

## Discussion

This study examined the longitudinal relation between lifestyle coaching and changes in the different types of motivation in generally obese participants of a CLI. For physical activity, changes in motivational regulation were fully in line with the tenets of SDT and MI: participants showed a shift towards a more autonomous type of motivation (i.e. controlled types of motivation decreased and autonomous types increased). Moreover, if participants perceived an autonomy supportive coaching style, this was generally found to predict a larger shift in autonomous types of motivation. As regards healthy dietary behaviour, however, except for a small decrease in external motivation, no favourable changes in different types of motivation were observed. The level of perceived autonomy supportiveness of the Lifestyle Coaches appeared not to have induced any positive changes. An important predictor of favourable changes in autonomous motivation was the intensity of the BeweegKuur programmes.

Our finding of an improvement in autonomous types of motivation for physical activity confirms the findings of previous studies that investigated the relation between autonomous motivation and lifestyle changes in interventions for energy balance related behaviours [28],[29]. The findings of our study specifically demonstrate that autonomy supportive lifestyle coaching in a ‘real world’ primary care CLI contributes to a favourable shift in motivational regulation for physical activity and that, in contrast, more controlled lifestyle coaching is related to higher amotivation and to a decrease of autonomous motivation.

However, in contrast to some previous studies [14],[29], we did not observe this favourable pattern of changes in quality of motivation for healthy dietary behaviour. It has been argued before that changing dietary behaviour may involve some physical and psychological discomfort, making it hard to be intrinsically motivated to do it [30]. Participants in our study had a higher level of controlled types of motivation for healthy diet, compared to physical activity at baseline. Moreover, almost 75% had losing weight as their main goal compared to 34% who chose health improvement as a goal. Previous studies revealed that higher controlled regulation of eating behaviours is related to poorer body image, lower psychological well-being [31], to a quantity focused eating regulation [32] and avoidance food planning [33]. Both strategies are negatively related to healthy eating behaviours [33]. Moreover it has been shown that physical appearance-focused (e.g. lose weight or gain a better physical appearance) instead of health-focused weight loss goals are related to less successful eating regulation strategies [30]. Furthermore, dietary behaviour may also include strong habits developed during childhood [34],[35]. Such findings demonstrate the complexity of eating behaviour regulation and indicate that a more autonomous motivation is indeed required for successful eating behaviour regulation. However, as we observed in our study, it also indicates that obtaining this autonomous motivation may be rather difficult.

Our finding that the influence of coaching on motivation for physical activity was virtually absent when programmes included physical activity guidance, seems in line with findings of van Hoecke et al. (2014) [36]. Provision of a physical activity programme may facilitate the need for competence and may therefore be equally effective as need supportive coaching [36]. Our findings in the samples that were stratified by programme type also indicate that the negative influence of perceived controlled coaching on autonomous motivation for healthy eating may be neutralised when an intervention includes a physical activity component. Previous studies have suggested a clustering of personal determinants of diet and activity [37] and it has been demonstrated that autonomous exercise motivation may ‘spill-over’ to facilitate improvements in eating self-regulation [38].

Except for the intrinsic motivation of participants in the Independent exercise programme, we found no relation between autonomy supportive lifestyle coaching and improvement of autonomous motivation for healthy diet. The absence of this relation may be caused by the LSC’s lack of knowledge about dietary behaviour and their insufficient skills to change it [39]. Previous failed attempts by participants to change their dietary behaviour may have resulted in a struggle and frustration with this behavioural goal, indicated by a lack of change in almost all types of the motivational pattern (“I want, I need *and* I must”). Failed attempts can cause frustration [40] and feelings of lack of competence, and would thus undermine one of the basic needs for autonomous motivation [41], if failures have repeatedly occurred. As a consequence the LSC should pay sufficient attention to improvement of feelings of competence.

The autonomy supportive coaching style we measured may not have fully covered the true breadth of autonomy support. Autonomy supportive coaching should include support of autonomy, competence and relatedness [16],[17]. The items included in our autonomy supportive coaching style questionnaire however mainly concerned autonomy. It has been demonstrated before that primary care nurses find it difficult to apply autonomy-supportive coaching in lifestyle related behaviours [42]–[44] and with communication about nutritional behaviour in particular [45]. Although participants in our study indicated that they were very satisfied with the performance of the LSC, they gave substantially lower scores for the LSC’s support to improve their dietary behaviour than the LSC’s support to improve their physical activity. Given the previously mentioned complex nature of unhealthy dietary behaviour [46], the LCSs must feature thorough knowledge of the problem and highly developed MI skills to favourably influence the autonomous motivation to improve this behaviour.

Although external regulation decreased on a group level, we observed a positive relation between autonomy-supportive coaching and an increase in external motivation for physical activity, which is not in accordance with SDT and MI. It has been observed before that people in treatment in general have a more external health locus of control [47] compared to those not in treatment. Moreover, our data revealed that participants judged the LSC to be very sympathetic and supportive. Participants with a higher external locus of control may have perceived the sympathy of the LSC as very rewarding, which may have induced an increase of their external motivation.

### Strengths and limitations

Strengths of the current study include its theoretical foundation, longitudinal design, real-life intervention setting and use of validated questionnaires. The self-selected sample used in the study limits its external validity. We used a self-report questionnaire completed by participants as a proxy measure of LSC performance. This is a relatively cheap and manageable way to measure professional performance in primary care. Nevertheless, the validity of this measure would be served by direct observations, or in the ideal case by standardized patients, the gold standard in measurement of professional performance. However, both these alternatives are time-consuming and costly, especially in studies with larger numbers of participants [48],[49].

Although this study was neither designed nor executed as an effectiveness trial, we performed an intention to treat analysis with the last observation carried forward as well as with the group mean imputation method [50] to replace missing data in order to address potential bias in the study results due to loss to follow up. The results of both approaches however did not change the key findings of our study regarding the predictive value of perceived coaching style on participants’ motivational regulation. However, it would require a randomized controlled trial to determine whether a change in coaching style would actually result in improved motivational regulation.

## Conclusion

The results of this study show that the motivation of participants of a facilitator-led CLI had changed towards a more autonomous motivation after 4 months of intervention, and that autonomy-supportive lifestyle coaching contributed to this change with respect to physical activity. A physical activity component in a CLI seems to be important for autonomous motivation for physical activity as well as for healthy dietary behaviour. Lifestyle coaching for healthy diet requires thorough knowledge about the problem of unhealthy dieting and solid coaching skills. The results indicate that LSC’s coaching skills were insufficient to shift the motivational regulation towards healthy diet in this generally obese population.

## Competing interests

The authors declare that they have no competing interests.

## Authors’ contributions

GMR carried out the study and wrote the manuscript, JJMM cooperated in the execution of the study and in the data analysis. MRCH cooperated in the study and provided data and FJMH assisted in the execution of the study. CV was a member of the project advisory group and SPJK conceived the concept of the study and cooperated in the execution. All co-authors critically reviewed and contributed to the manuscript. All authors read and approved the final manuscript.

## Additional file

## Supplementary Material

Additional file 1: Items per type of coaching. Click here for file
