# Supplementary material for: The contribution of lifestyle coaching of overweight patients in primary care to more autonomous motivation for physical activity and healthy dietary behaviour: results of a longitudinal study
Source: Int J Behav Nutr Phys Act. 2014 Jul 16;11:86. doi: 10.1186/s12966-014-0086-z (PMC4132211; doi:10.1186/s12966-014-0086-z)
Supplement: Additional file 1: — Items per type of coaching. [file s12966-014-0086-z-S1.docx]

Additional file 1: Items per type of coaching

| Autonomy supportive | The LSC   - made me realize that participating in the BeweegKuur is important for me - made me aware that my level of physical activity is low - made me realize that my overweight is related to my lifestyle - made me see the importance of the BeweegKuur which made me decide to participate - clearly informed me what participation means - took me very serious in our conversations   My conversations with the LSC have taken away all my doubts about participating in the BeweegKuur |
| --- | --- |
| Controlled | The LSC   - decided for me to participate in the BeweegKuur - hardly listened to me during our conversations |
| Protocol adherent | The LSC   - performed body measurements (BMI, waist circumferemce) - assessed my level of physical activity - referred me to the dietician - assessed my psychological well being - measured my blood values - referred me to the physical therapist |
